# Investigation of Possible Cardiac Manifestations of Dysautonomia in a Patient With Acquired Ondine’s Curse Syndrome: A Rare Clinical Case Report

**DOI:** 10.7759/cureus.102760

**Published:** 2026-01-31

**Authors:** Stefanos Votsis, Konstantinos Marmagkiolis

**Affiliations:** 1 Cardiology, Medical College of Georgia at Augusta University, Augusta, USA; 2 Cardiology, 424 Military Hospital, Thessaloniki, GRC; 3 Cardiology, University of South Florida, Tampa, USA

**Keywords:** acquired central hypoventilation syndrome, arrythmia, dysautonomia, ondine's curse, ventrolateral medulla stroke

## Abstract

Ondine's curse, also known as central alveolar hypoventilation syndrome, is a very rare condition characterized by failure of breathing mechanisms during sleep. The most usual cause of acquired Ondine's curse syndrome is a stroke of the brainstem, causing dysautonomia, which is primarily manifested by sleep apnea, but can also cause cardiac rhythm disturbance. We present the case of an 81-year-old female with a history of a brainstem and cerebellum ischemic stroke one month ago, which resulted in acquired Ondine's curse syndrome. The patient had been sleeping with respiratory support at a rehabilitation center and was transferred to our hospital in order to investigate possible arrhythmias due to the concurrent dysautonomia. The patient was hospitalized and continuously monitored at our hospital for a total of 72 hours, during which many episodes of sinus bradycardia were monitored. These episodes occurred only when the patient was asleep and in tandem with prolonged sleep apnea episodes. It is unclear whether bradycardia was within the normal circadian cardiac rhythm pattern or was a manifestation of dysautonomia. Arrhythmiologic (electophysiology) consultation deferred further treatment or monitoring measures due to a lack of clear indications.

## Introduction

Ondine's curse, which is also known as central alveolar hypoventilation syndrome, is a very rare syndrome characterized by inadequacy of the cerebral breathing center during sleep. Affected patients have a normal breathing pattern when they are awake, but inevitably progress to extended apnea after falling asleep [1]. The syndrome can be either congenital (which is the more usual presentation) or acquired, which is extremely rare. The most usual cause of acquired Ondine's curse syndrome is a stroke of the brainstem, and particularly the ventrolateral medulla, causing dysautonomia, which is primarily manifested by sleep apnea, but can also cause cardiac rhythm disturbance [2].

Laboratory criteria for the diagnosis of Ondine's curse are not well established [3]. Some authors propose that prolonged and persistent periods of apnea associated with desaturation and hypercapnia during non-rapid eye movement (REM) sleep should be demonstrated for diagnosis, while other ones mention some criteria such as: (a) normal pulmonary and mediastinal anatomy, (b) pO2 normalization through voluntary breathing when awake and (c) precipitation of alveolar hypoventilation with diminished voluntary control (i.e., sleep) [4].

## Case presentation

Our patient was an 81-year-old female with a history of a brainstem and cerebellum ischemic stroke one month ago, which had resulted in acquired Ondine's curse syndrome and anemia. The patient had been sleeping with respiratory support at a rehabilitation center, and O2 pulse oximetry revealed prolonged episodes of bradycardia during sleep. She was transferred to our hospital mainly because the rehabilitation center a) lacked cardiac monitoring and Holter capacities, and b) in order to receive immediate pacemaker implantation as a supportive measure if bradycardia was confirmed and a pertinent diagnostic indication surfaced.

The patient was hospitalized and continuously monitored in a hospital unit with such capability for a total of 72 hours in order to reach a diagnosis. Indeed, during her stay, many episodes of sinus bradycardia were monitored. These episodes occurred only when the patient was asleep. The minimum heart rate was 45 beats per minute and occurred concomitantly with the patient's episodes of sleep apnea, during which the patient's respiratory function was assisted mechanically. Arrhythmiology (electrophysiology) consultation also took place during her hospital stay. Pacemaker implantation was deferred due to the fact that the patient remained hemodynamically stable during the aforementioned bradycardia episodes (blood pressure of 110/70 mmHg, mean arterial pressure (MAP) of 90 mmHg). The patient returned to the rehabilitation center for further treatment, and, after two months in total, was discharged home.

## Discussion

Ondine’s curse syndrome was initially discovered and described by Kumral et al. in patients who had undergone bilateral spinothalamic tract cordotomies and who had preserved their voluntary respiratory function but manifested inadequate response to inhaled carbon dioxide [5]. It owes its unusual name to a Nordic legend, according to which a maiden named Ondine curses her cheating fiancé to never be able to sleep again, because if he does, he will stop breathing and die.

In the acquired form of the syndrome, damage at three different areas in the brainstem is known to disrupt respiratory control neurons and cause hypoventilation: a) the dorsal respiratory group (which is mainly inspiratory), which is located in the dorsal medulla within the nucleus tractus solitarii (NTS); b) the ventral respiratory group, which is a column of inspiratory, expiratory and rhythm-generating neurons extending from the first segment of cervical spinal cord to below the facial nuclei; c) the parabrachial/Kölliker-Fuse complex, which can be found at the dorsolateral upper pons, which is responsible for the alteration of inspiration and expiration. Moreover, the NTS is the encephalic area primarily responsible for cardiorespiratory control, receiving afferent information from arterial baroreceptors and cardiac receptors. It regulates cardiac rate and vascular pressure with diverse efferent projections to the medulla and pons. This means that damage to its neurons can potentially cause cardiac arrhythmias [2]. Of note, there is a case report of acquired Ondine's curse as a result of brainstem ischemic injury, which resulted in cardiopulmonary arrest, although whether the aforementioned injury is the cause or the result of the arrest is open for debate [6].

In our case, the patient was continuously monitored for 72 hours, and transient sinus bradycardia was confirmed. The fact that the aforementioned arrhythmia appeared only when the patient was asleep a) meant that no pertinent symptoms could be reported by the patient and b) would permit the hypothesis that Ondine's curse syndrome was its cause. This argument may be validated by the fact that the bradycardia episodes occurred at the same time that the patient's respiratory function required mechanical support by a ventilator. On the other hand, it is common knowledge that bradycardia episodes may occur even in healthy individuals during sleep, and they are not a dysautonomia manifestation, but rather a normal effect of the autonomic nervous system within the normal circadian rhythm limits. Be that as it may, because of the bradycardia's relative mildness (45 beats per minute was the minimum heart rate recorded), further therapeutic or monitoring measures were considered unnecessary. Further autonomic testing (e.g., the tilt test), although pertinent as well, might not have been a useful diagnostic tool, since the bradycardia episodes were all sleep-related. It may also be noted that a third possibility (bradycardia due to vagal reflex secondary to apnea) was not considered probable due to the fact that our patient was connected to a respiratory assistance device during sleep and therefore apnea never occurred, nor was it possible. For the exact same reason, our patient never exhibited hypoxia or hypercapnia, which otherwise might have been considered as possible causes of bradycardia.

Several limitations should be acknowledged in our case. It should be noted that the diagnostic approach lacked formal polysomnography (PSG) with simultaneous 12-lead ECG, which might have provided a more precise correlation between sleep stages, respiratory events, and rhythm changes. Moreover, there are obvious, inherent limitations in a single-case retrospective observation in establishing a causal link between brainstem lesions and specific arrhythmias, added to the limitation of sparse similar bibliographic data due to the rarity of acquired Ondine's curse syndrome.

## Conclusions

Acquired Ondine's curse is an extremely rare syndrome that develops after the brainstem, particularly the ventrolateral medulla, is damaged by a traumatic, ischemic, infectious, or neoplastic event. It primarily results in central hypoventilation, which displays itself with apnea episodes during sleep. It can also cause dysautonomia, which might manifest in cardiac rhythm abnormalities. Healthcare providers should maintain a high suspicion threshold for cardiac arrhythmias, monitor these patients accordingly, and take therapeutic measures should pertinent indications occur. However, the usual diagnostic algorithms may not always be useful and might not provide the needed diagnostic explanation, especially in extremely rare syndromes, cases, and presentations, where many factors (including limited bibliography) may cause uncertainty. In such circumstances, a case-by-case assessment is the most prudent modus operandi for the medical team.
